# F164. VENTRAL AND DORSAL STRIATAL DYSFUNCTION DURING REWARD ANTICIPATION IS ASSOCIATED WITH NEGATIVE SYMPTOMS IN PATIENTS WITH SCHIZOPHRENIA AND HEALTHY INDIVIDUALS

**DOI:** 10.1093/schbul/sby017.695

**Published:** 2018-04-01

**Authors:** Marta Stepien, Andrei Manoliu, Roman Kubli, Karoline Schneider, Philippe N Tobler, Erich Seifritz, Marcus Herdener, Stefan Kaiser, Matthias Kirschner

**Affiliations:** 1 Psychiatric Hospital, University of Zurich; 2 Laboratory for Social and Neural Systems Research, University of Zurich; 3 Geneva University Hospitals

## Abstract

**Background:**

Negative symptoms are a core feature of schizophrenia and also found in healthy individuals in subclinical forms. According to the current literature the two negative symptom domains, apathy and diminished expression may have different underlying neural mechanisms. Previous observations suggest that striatal dysfunction is associated with apathy in schizophrenia. However, it is unclear whether apathy is specifically related to ventral or dorsal striatal alterations. Here, we investigated striatal dysfunction in patients with schizophrenia and a non-clinical population, to determine whether it is a relevant neural correlate for apathy.

**Methods:**

Chronic schizophrenia patients (n= 16) and healthy controls (n=23) underwent an event- related functional MRI, while performing a variant of the Monetary Incentive Delay Task. The two negative symptom domains were assessed in both groups using the Brief Negative Symptoms Scale.

**Results:**

In schizophrenia patients, we saw a strong negative correlation between apathy and ventral and dorsal striatal activation during reward anticipation. In contrast, there was no correlation with diminished expression. In healthy controls, global negative symptoms were correlated with decreased dorsal striatal activity.

**Discussion:**

This study replicates our previous findings of a correlation between ventral striatal activity and apathy but not diminished expression in chronic schizophrenia patients. The association between apathy and reduced dorsal striatal activity suggests that impaired action-outcome selection is involved in the pathophysiology of motivational deficits. Finally, our findings in healthy controls support the idea that striatal alterations are a plausible neural correlate for negative symptoms in both a clinical and a subclinical context.

